# High Temperature Deformation Behavior of In-Situ Synthesized Titanium-Based Composite Reinforced with Ultra-Fine TiB Whiskers

**DOI:** 10.3390/ma11101863

**Published:** 2018-10-01

**Authors:** Rongjun Xu, Bin Liu, Yong Liu, Yuankui Cao, Wenmin Guo, Yan Nie, Shifeng Liu

**Affiliations:** 1 State Key Lab of Powder Metallurgy, Central South University, Changsha 410083, Hunan, China; xurongj@csu.edu.cn (R.X.); yonliu@csu.edu.cn (Y.L.); caoyuankui@csu.edu.cn (Y.C.); 2 College of Mechanical and Energy Engineering, Shaoyang University, Shaoyang 422000, Hunan, China; wenminguo@hotmail.com; 3 YuanMeng Precision Technology (Shenzhen) Institute, Shenzhen 518000, Guangdong, China; nieyan725@163.com; 4 College of Metallurgy Engineering, Xian University of Architecture & Technology, Xi’an 710055, Shanxi, China

**Keywords:** titanium based composites, hot deformation, processing map, ultra-fine TiB, powder metallurgy

## Abstract

A TiB/Ti-6Al-4V composite reinforced with ultra-fine TiB whiskers (UF-TiB) was prepared by the powder metallurgy method. High temperature compression tests were carried out to study the hot deformation behavior of the UF-TiB/Ti-6Al-4V composite. The compressive deformation was performed in the temperature range of 900–1200 °C and the strain rate range of 0.001–10 s^−1^. The results showed that stable flow occurred at the condition of 900–1200 °C/0.001–0.01 s^−1^. The optimum working condition was 900 °C/0.001 s^−1^, with the deformation mechanism of dynamic recrystallization (DRX). Instable flow occurred when the strain rate was higher than 0.01 s^−1^, where the failure modes included adiabatic shear deformation, whisker breakage and whisker/matrix debonding. The deformability of the UF-TiB/Ti-6Al-4V composite was much better than the traditional casted and the pressed + sintered TiB/Ti-6Al-4V composites, which are typically reinforced with coarse-grained TiB whiskers. The high deformability was primarily attributed to the ultra-fine reinforcements, which could coordinate the deformation more effectively. In addition, a fine matrix microstructure also had a positive effect on deformability because the fine matrix microstructure could improve the grain boundary sliding.

## 1. Introduction

Titanium alloy reinforced with fibers or particles, also known as titanium matrix composites (TMCs) are commonly used in aerospace and marine industries that require their high specific strength and good corrosion resistance [1,2,3]. Generally, TiC, SiC and TiB are often used as reinforcing phases in titanium alloys, and they indeed improved the performance of titanium alloys to some extent [2,4]. In particular, the titanium boride (TiB) whisker is an effective reinforcement for titanium alloys, since the ceramic TiB phase has a high strength and an excellent thermal stability. Previous studies have indicated that the mechanical performances of titanium alloy are significantly enhanced with the addition of TiB whiskers [5,6].

Hot deformation is not only an indispensable step for the manufacturing process, but also an efficient method to control and enhance the mechanical properties of TMCs [7]. Previous literature has stated that the hot deformation reaction and subsequent microstructure evolution of TMCs are significantly influenced by the presence of TiB [8]. Furthermore, titanium matrix and reinforcement are apparently different in elastic modulus, which may cause the deformation incoordination during the plastic deformation process. Thus, the composites crack easily and their deformation ability becomes seriously degraded. Many researchers have studied the hot deformation behavior of titanium alloys reinforced with TiB whiskers. Cortazar et al. [9] investigated the effect of TiB on deformability of the TiB/Ti-6Al-4V composite prepared by the cast method. The results showed that the as-casted Ti-6Al-4V alloy could be well deformed at 870 °C. However, with the additions of 8.9 vol. % TiB, the stable deformation of TiB/Ti-6Al-4V composite could only occur at 1010 °C. Bhat et al. [10] prepared the Ti6Al4V-2.9B composite via conventional blended element powder metallurgy (P/M) method and studied the thermomechanical response of the composite. The composite had a matrix grain size of ~15 μm, a TiB length of 30–50 μm and a TiB diameter of 5–8 μm. The unstable deformation occurred in the *α* + *β* phase field (950–1000 °C) over the full range of strain rates and occurred at higher strain rates (1–10 s^−1^) over a broad range of temperature in the *β* phase field (>1000 °C). The main failure mode of the composite was the fracture of TiB and the debonding at the TiB/Ti-6Al-4V interface. Huang et al. [11] also studied the hot compression characteristics of TiB/Ti-6Al-4V composites with 5 vol. % TiB. The composite had a matrix grain size of ~20 μm, a TiB length of 20–30 μm and a TiB diameter of 2–5 μm. The results showed that the unstable deformation occurred at high strain rates (1–10 s^−1^) between 900 and 1100 °C. The unstable characteristics of the composite could be expressed as debonding, cracking and flow localization bands. The above composites all have a coarse matrix and reinforcement, and their deformability is limited, especially in the *α* + *β* phase field. Refining matrix grain size and reinforcement size may improve the deformability of the composites. However, the hot deformation behavior of TMCs reinforced with ultra-fine TiB whiskers (UF-TiB/Ti-6Al-4V) has rarely been reported. Further research of the hot deformation behavior of TMCs reinforced with ultra-fine reinforcement has an important guiding significance for the processing of titanium matrix composites.

There are numerous methods for preparing titanium matrix composites [12,13]. Particularly, the spark plasma sintering (SPS) method is capable of obtaining an in-situ synthesis of TiB reinforcement, where TMCs with a fine matrix structure and ultra-fine reinforcements can be obtained [14,15,16]. In this study, TiB/Ti-6Al-4V composite reinforced with ultra-fine TiB whiskers was prepared by the SPS method and the hot deformation behavior of UF-TiB/Ti-6Al-4V composite was studied. The processing map was built to evaluate its hot deformation capability and the damage mechanism is discussed below.

## 2. Materials and Methods

The UF-TiB/Ti-6Al-4V composite was prepared by mixing Ti-6Al-4V alloying powder, Ti powder and TiB_2_ powder (TiB_2_ was used to in-situ react with Ti to form TiB), and spark plasma sintering. The raw material powders used in this study were supplied by Northwest Nonferrous Metals Research Institute, (Xi’an, China). Table 1 lists the physical characteristics of the raw powders. The analysis of the raw powders was conducted on a Laser Particle Size Analyzer (Mastersizer, 3000, Malvern Panalytical, Cambridge, UK) and a Nitrogen/Oxygen Analytical Instrument (LECO TCH600, LECO, St. Joseph, MI, USA). The raw powders were mixed using a V-type mixer (Zgsujia, V-20, Yupan, Nanjing, China) for two hours. Argon was used as a protective atmosphere during the mixing process to prevent oxidation. The as-mixed powders were subjected to spark plasma (FCT D25/3, FCT Systeme GmbH, Rauenstein, Germany) sintering at 1373 K for 10 min, with a pressure of 40 MPa.

The cylindrical samples with a diameter of 8 mm and a height of 12 mm were cut from the spark plasma sintered bulk via wire electrical discharge machining (WEDM, DK35, Huazheng, Guangzhou, China) with a cutting speed of 60 cm^2^/min. Surfaces of the cylindrical samples were ground and polished with 500–2000 grid SiC paper. Uniaxial compression tests were performed in vacuum (1 × 10^−2^ Pa) at 900, 1000, 1100 and 1200 °C, applying a computer-aided testing equipment (GLEEBLE, 3500, DSI, New York, NY, USA). The strain rate of the compression tests was set at 0.001, 0.01, 0.1, 1 and 10 s^−1^. The samples were heated to the given temperature at a heating rate of 5 K/s and held for 5 min prior to compressing. After deformation, the compressed samples were quenched to room temperature in water. Due to the friction between the samples and the instrument, barreling occurred during the high temperature compression test. During the deformation, local overheating due to adiabatic shear deformation may occur at the higher strain rate. Thus, the influence of the friction and the local overheating on stress-strain curves were compensated by the method provided by [17]. The deformed samples were ground and polished before being observed by a scanning electron microscope (SEM, Nova NanoSEM230, FEI, Hillsboro, OR, USA). Phase analysis was conducted at room temperature using an X-ray diffractometer (XRD, Max255Ovb+, RigakuD, Tokyo, Japan) with a Cu/K*_α_* resource.

## 3. Results

### 3.1. Initial Microstructure of the UF-TiB/Ti-6Al-4V Composite

**Figure 1:**
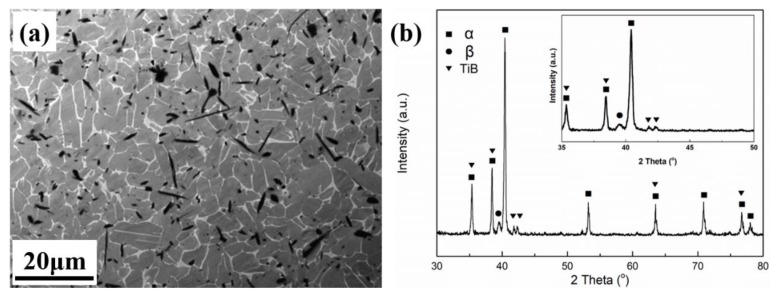
Microstructure and X-ray diffractometer (XRD) patterns of the UF-TiB/Ti-6Al-4V composite: (**a**) backscattered electron image; (**b**) XRD patterns.

The microstructure and XRD patterns of the spark plasma sintered UF-TiB/Ti-6Al-4V composite are shown in Figure 1. The composite consisted of a grey phase, a bright phase and a dark phase. According to the XRD patterns from Figure 1b, the grey phase can be identified as the *α* phase, the bright phase as the *β* phase and the dark phase as the TiB whisker, respectively. In our previous work [16], it was reported that the Gibbs free energy of the Ti and B reaction to form TiB was −157 KJ/mol. The initial and completed reaction temperatures between Ti and TiB_2_ during spark plasma sintering were approximately 800 °C and 1150 °C. The TiB_2_ could be completely converted to TiB at 1150 °C under the condition of adding enough titanium powder. The zoomed in XRD data between 35° and 50° are also shown in the upper right corner of Figure 1b. There was no diffraction peak of TiB_2_ presented, indicating that the TiB_2_ had completely reacted with the Ti matrix to form TiB whiskers. The TiB whiskers with random orientation were uniformly distributed on the Ti6Al4V matrix. The grain size of the matrix was approximately 5–12 μm, the length of the TiB whiskers was approximatelr 5–20 μm, and the diameter was 0.1–2 μm. The matrix grain size and the whisker size were both smaller than that of the TiB/Ti-6Al-4V composite prepared by the conventional pressed + sintered method, where the matrix grain size was typically longer than 15 μm, the length of the TiB whisker reached up to 30–50 μm, and the diameter of the TiB was approximately 5–8 μm [10].

The size of the TiB whisker in this composite was estimated based on the diffusion thermodynamics. The growth of TiB was conducted according to the calculation reported by Fan et al. [18].

(1) $$x=k\sqrt{t},$$

where *x* and *k* represent the length and the growth rate of the whiskers, respectively, and *t* represents the time in second. The relationship between the growth rate of TiB and the temperature can be expressed as follows:

(2) $$k=k_{0}\exp(-\frac{Q_{K}}{2RT}),$$

where *k*_0_ is a constant representing the frequency factor, *Q_K_* represents the activation energy for nucleation of the TiB, and *T* and *R* are the temperature in Kelvin and the gas constant, respectively. The value of *k*_0_ and *Q_K_* are 17.07 × 10^−4^ m/s^1/2^ and 190.3 kJ mol^−1^, respectively [18]. The computed *k* value at 1100 °C was 4.09 × 10^−6^ m/s^1/2^. The estimated length of the TiB whisker sintering for 10 min was calculated approximately 10 μm, which was close to the average length (12 μm) of the TiB whisker in this study. This suggested that the Ti powders had reacted completely with the TiB_2_ powders.

### 3.2. Hot Deformation of the UF-TiB/Ti-6Al-4V Composite

**Figure 2:**
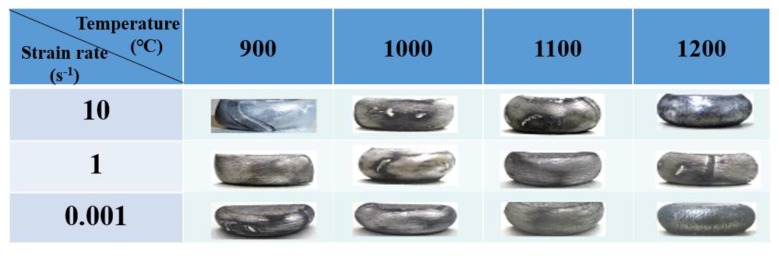
Side views of the UF-TiB/Ti-6Al-4V composite deformed at various temperatures and strain rates.

**Figure 3:**
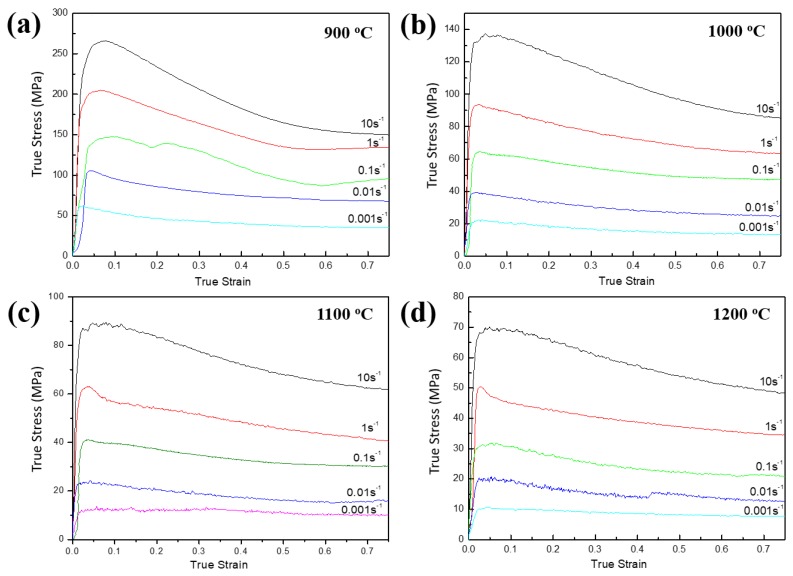
True stress-true strain curves of the UF-TiB/Ti-6Al-4V composites after being compensated for the influence of local overheating and friction: (**a**) 900 °C, (**b**) 1000 °C, (**c**) 1100 °C and (**d**) 1200 °C.

Figure 2 shows the side views of the UF-TiB/Ti-6Al-4V composite deformed at various temperatures and strain rates. No cracks could be observed, which suggested that the UF-TiB/Ti-6Al-4V composite had a good deformability. The typical flow curves of the UF-TiB/Ti-6Al-4V composite deformed at various temperatures and strain rates are shown in Figure 3. The flow stress increased with the increase of strain rate and the decrease of temperature. The flow stress slowly decreased after they reached a peak stress at 900 °C and 1000 °C (Figure 3a,b). At 1100 °C and 1200 °C (Figure 3c,d), the continuous flow softening behavior was observed at the higher strain rates (1–10 s^−1^). The stable flow was observed after a peak stress at the lower strain rates (0.1–0.001 s^−1^). This indicated the steady states of deformation. The microstructural evolution during the deformation process led to a different trend of true stress-true strain curve. The continuous flow softening indicated the mechanisms like flow localization or unstable flow because of the micro-cracks. The steady flow at a relative high temperature could be attributed to the dynamic recovery (DRV) or the dynamic recrystallization (DRX) [19]. These assumptions had to be proven and are discussed in Section 3.4.

**Figure 4:**
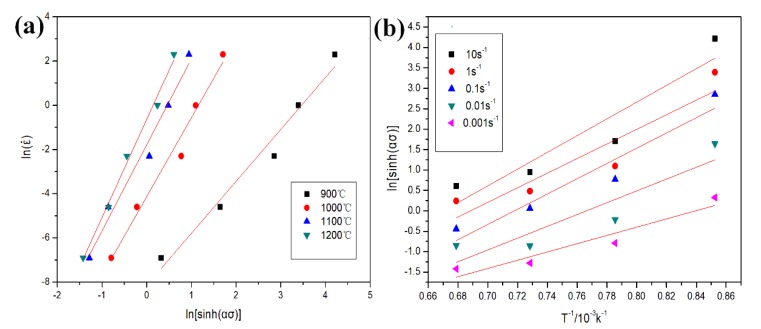
(**a**) Arrhenius plot of ln*ε* versus ln(sinh(*ασ*)); (**b**) the Arrhenius plot of ln(sinh(*ασ*)) versus 1/*T*.

The Arrhenius equation is widely used to describe the relationship between flow stress and temperature as well as strain rate during hot deformation [20]:

(3) $$\varepsilon =A{\left[\sinh(\alpha \sigma )\right]}^{n}\exp[-Q/(RT)],\;$$

where *ε* is the strain rate, *A* is a material constant, *σ* is the flow stress, *n* is the stress exponent, *Q* is the activation energy, *R* is the gas constant, and *T* is the absolute temperature. Figure 4a,b show the Arrhenius plot of ln *ε* versus ln(sinh(*ασ*)) and the plot of ln(sinh*(ασ*)) versus the 1/*T*, respectively. Figure 4a shows the variation of flow stress with strain rate at different temperatures. The slope of ln(sinh(*ασ*)) versus 1/*T* was used to calculate the activation energy for hot deformation (*Q_HD_*) to be 410.77 KJ mol^−1^. The value of *Q_HD_* was higher than the self-diffusion (*Q_SD_*) of the *α* phase [21]. Braga et al. [22] obtained similar results in the Ti-6Al-4V alloy, where they stated that the high value of *Q_HD_* resulted from the point of dynamic recrystallization. Briottet et al. [23] found that the high value of the *Q_HD_* could be attributed to the difference in deformation ability between the *β* phase and the *α* phase during hot deformation. The movement of the *β* phase was limited by *α* phase. This resulted in the movement of matrix mainly dominated by the deformation of the *α* phase. Furthermore, the fine TiB whiskers could hinder the slide of dislocations and the migration of grain boundaries during the deformation, which would further increase the value of *Q_HD_*.

### 3.3. Processing Map of the UF-TiB/Ti-6Al-4V Composite

The flow stress data were used to further analyze the constitutive behavior of the UF-TiB/Ti-6Al-4V composite by using the processing map. The dimensionless parameter called power dissipation efficiency was defined as follows [24]:

(4) $$\eta =\frac{2m}{m+1},$$

where *m* represents the strain rate sensitivity coefficient, which indicated that the consumption of input power through deforming rather than heat dissipating. The *m* value at a given temperature and strain was defined as follows:

(5) $$m=\frac{\partial \log\sigma }{\partial \log\overset{•}{\varepsilon }},$$

The power dissipation map was composed of various *η* values at different deformation temperatures and strain rates. Based on the extremum principle of irreversible thermodynamics, the instability criterion that was applied to the large plastic flow was given by Equation (6) [25].

(6) $$\xi (\varepsilon )=\frac{\partial \ln(m/(m+1))}{\partial \ln\varepsilon }+m<0,$$

**Figure 5:**
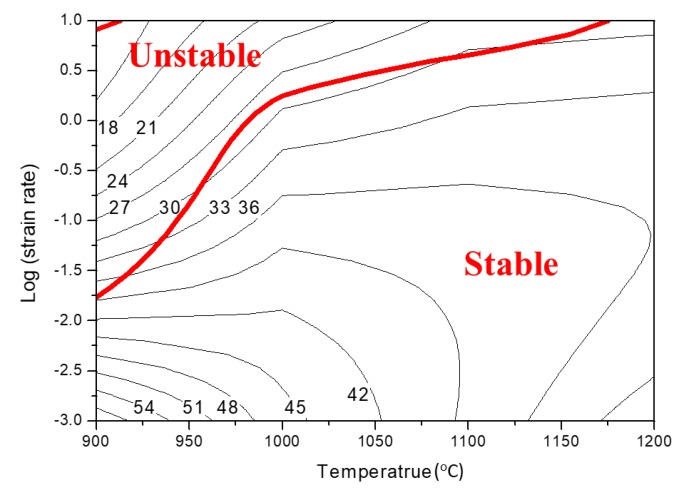
Processing map of the UF-TiB/Ti-6Al-4V composite prepared by P/M method (*ε* = 0.6)

The processing map developed for UF-TiB/Ti-6Al-4V composite at a true plastic strain of 0.6 was obtained through overlaying the instability map on a power dissipation map (Figure 5). The contour numbers represented the values of *η* which increased as the temperature and strain rate decreased. The processing map exhibited a convergent domain between 900 °C and 1200 °C and a strain rate range between 0.001 s^−1^ and 0.01 s^−1^ with a peak *η* of 58% at 900 °C/0.001 s^−1^, which could be caused by the DRX of the matrix. According to Equation (6), as shown in the unstable area in Figure 5, the flow instability occurred when the strain rate was higher than approximately 0.01 s^−1^. The unstable flow of the UF-TiB/Ti-6Al-4V composite at high strain rates could be caused by the appearance of adiabatic shear bands and local flow. The processing map of the UF-TiB/Ti-6Al-4V composite had a similar tendency to the conventional TiB/Ti-6Al-4V composite prepared by the pressed + sintered method [10] and the casting method [26]. The unstable area in the processing map of the UF-TiB/Ti-6Al-4V composite was obviously smaller than the pressed + sintered and the casted TiB/Ti-6Al-4V composites, which indicated that the UF-TiB/Ti-6Al-4V composite had a better deformability. In addition, local stress concentration played an important role in the failure mechanism of the composite and simply relying on the processing map was not enough to determine the failure mechanism of the material. A further investigation of the microstructure after deformation was conducted, and the results are reported in the next section.

### 3.4. Deformed Microstructures

**Figure 6:**
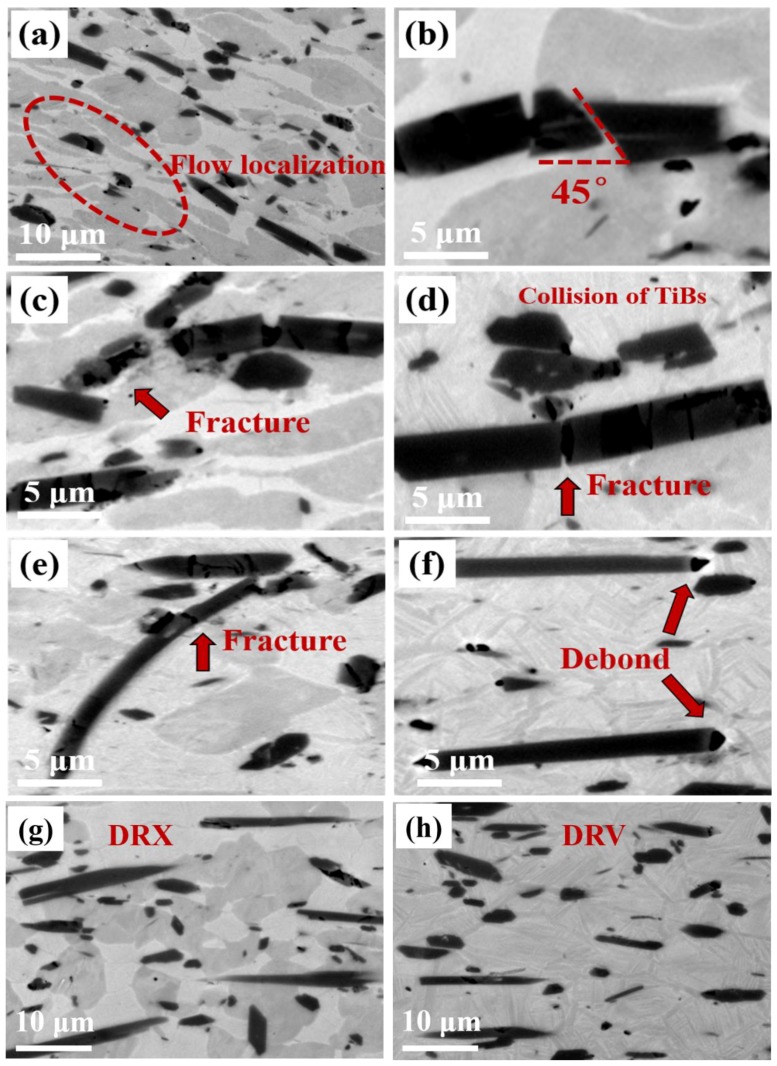
Microstructures of the UF-TiB/Ti-6Al-4V composite deformed at different conditions: (**a**) 900 °C/10 s^−1^, (**b**,**c**) 900 °C/1 s^−1^, (**d**,**e**) 1000 °C/10 s^−1^, (**f**) 1100 °C/10 s^−1^, (**g**) 900 °C/0.001 s^−1^, (**h**) 1100 °C/0.001 s^−1^.

The microstructures of UF-TiB/Ti-6Al-4V composites compressed at different temperatures and strain rates are shown in Figure 6. The microstructures obtained were sensitive to deformation temperature and strain rate. Several failure modes of the composite are observed in the images. Figure 6a shows the microstructure of specimen deformed at 900 °C/10 s^−1^. The flow localization appeared along the shear direction, which revealed the occurrence of adiabatic shear deformation [27]. The formation of shear bands was caused by the adiabatic conditions during the high strain rate deformation and the low thermal conductivity of the titanium based composite. Figure 6b,c show the microstructure of specimens deformed at 900 °C/1 s^−1^. Fractures of the reinforcement were observed along the shear direction, that is, 45° to the compression direction (Figure 6b). Typically, such soft oriented whiskers are coordinated with the deformation of matrix, and shear fractures occur under compressive stress. Figure 6d,e show the microstructure of specimen deformed at 1000 °C/10 s^−1^, where the TiB whiskers collided with each other during deformation, and caused the TiB fracture. When the temperature was increased to 1100 °C, the amount of whisker fracture decreased, as shown in Figure 6f. Only cavitation can be observed at the matrix/TiB interfaces. In summary, three types of failures formed during deformation: fractures caused by reinforcement collision, fractures caused by shear deformation and debonding at high temperature.

**Figure 7:**
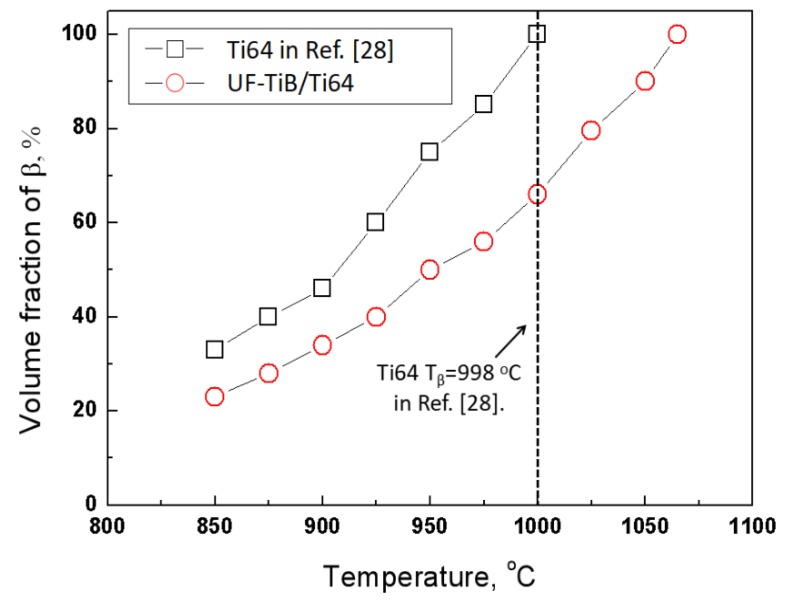
Temperature dependent variation of the *β* vol. % in both the UF-TiB/Ti-6Al-4V composite and the conventional Ti-6Al-4V alloy in Ref. [28].

Figure 6g,h show the microstructure of specimens deformed at 900 °C/0.001 s^−1^ and 1100 °C/0.001 s^−1^. No whisker fractures or cavitation can be observed. The DRX occurred at 900 °C/0.001 s^−1^ (Figure 6g), resulting in a fine and equiaxed microstructure. When deformed at 1100 °C, i.e., the temperature above the *β* transus, DRV occurred and grains grew very clearly (Figure 6h). Combined with the microstructure observation and processing map, it can be speculated that the highest value of *η* in the *α* + *β* phase field was caused by the DRX of the matrix. In addition, Figure 7 shows the fluctuating volume fraction of the *β* phase of the Ti-6Al-4V alloy in Ref. [28] and the UF-TiB/Ti-6Al-4V composite in this study. The *β* transus of the Ti-6Al-4V alloy was approximately 998 °C, which was approximately 70 °C lower than that of the UF-TiB/Ti-6Al-4V composite. This indicated that the addition of element B might increase the beta transus. Similar results were also observed in previous literature [29], where it was suggested that the mechanism for the increase of the beta transus could be attributed to the supersaturated B atoms in solid solution.

## 4. Discussion

### 4.1. Damage Mechanism of the UF-TiB/Ti-6Al-4V Composite

During the deformation of metal matrix composite, the reinforcements will be deformed with the matrix if they are deformable. If the reinforcements do not deform, strain incoordination between the matrix and the reinforcements is a possible source for the void formation. In this study, because of the significant differences in the elastic modulus between the Ti-6Al-4V matrix and the TiB whiskers, stress concentrations between the matrix and the whiskers were produced. This would result in separation of whiskers from the Ti matrix or the whiskers cracking when the bond strength of the whisker/matrix was lower than the local stress and the strength of whiskers. As the cavities grew and aggregated, this eventually led to material damage. Cherukuri [30] derived a calculation model for the prediction of fiber/whisker failure of metal matrix composite. Assuming that the shear stress in fiber/matrix interface was equal to the fiber strength, the critical length of fiber where the fracture occurred was estimated by the following equation:

(7) $$\sigma_{fiber}*\frac{\pi d^{2}}{4}=\tau_{0}\pi d\frac{l}{2},$$

where *l* is the critical length of the fiber, *d* is the diameter of the fiber, *τ*_0_ is the shear stress in fiber/matrix interface, and *σ_fiber_* is the yield strength of the fiber. 

Assuming that:

(8) $$\tau_{0}=\sigma_{matrix}/2,$$

Thus, the critical relationship between the aspect ratio of the fibers (*l/d*), the strength of the fibers (*σ_fiber_*) and the strength of the matrix (*σ_matrix_*) can therefore be derived as follows:

(9) $$\frac{l}{d}=\frac{\sigma_{fiber}}{\sigma_{matrix}},$$

According to Equation (9), the fiber would undergo transverse fracture if the aspect ratio of fiber exceed the ratio of the strength of the fiber and the matrix ($\frac{l}{d}>\frac{\sigma_{fiber}}{\sigma_{matrix}}$). In contrast, when the aspect ratio of the fibers was lower than the ratio of the strength of the fiber to the matrix ($\frac{l}{d}<\frac{\sigma_{fiber}}{\sigma_{matrix}}$), cavitation at the matrix/fiber interfaces would occur. 

In this composite, the *σ_matrix_* was relatively high in the *α* + *β* phase field, which could make the aspect ratio of the whisker exceed the ratio of the strength of the whisker and the matrix, where the whisker underwent a transverse fracture, as shown in Figure 6a–e. In contrast, increasing the deformation temperature would increase the volume fraction of the *β* phase. Due to the BCC *β* phase being soft at high temperature, the stress could be more effectively released by the plastic deformation of the matrix. Thus, the lesser stress transferred to the whiskers would not be enough to cause the crack of whiskers, but it would cause cavitation at the TiB/matrix interfaces, as shown in Figure 6f. Thus, the various matrix strengths in the various phase fields could play an important role in the damage mode of particles during the isothermal deformation.

### 4.2. Improved Deformability of the UF-TiB/Ti-6Al-4V Composite

The stable flow area in the processing map of the UF-TiB/Ti-6Al-4V composite was larger than those of the conventional pressed + sintered Ti-6Al-4V-2.9B [10] and casted [26] Ti-6Al-4V-0.55B composites, which visually confirmed that the UF-TiB/Ti-6Al-4V had a good deformability. Besides, the Ti-6Al-4V-0.55B composite also appeared in a second instability zone due to the fracture and debonding of reinforcement near the phase transition temperature. Although the B (B = 2 wt. %) content in this study was higher, this phenomenon was not observed in this UF-TiB/Ti-6Al-4V composite. The higher deformability could result from the fine-grained Ti-6Al-4V matrix and the ultra-fine TiB reinforcement. The conventional pressed + sintered TiB/Ti-6Al-4V composite had a matrix grain size of ~15 μm, a TiB whisker length of 30–50 μm and a TiB whisker diameter of approximately 5–10 μm. The authors in Ref. [26] did not give the initial grain size of the casted TiB/Ti-6Al-4V composite, but according to its preparation method, it seems that the microstructure must be bigger than that used in this study. The coarse microstructure of the matrix reduced the slide ability of the grain boundary and limited its deformation. The coarse TiB reinforcements made it difficult to coordinate the deformation between the matrix and the reinforcements. In this study, the adoption of spark plasma sintering technology allowed the synthesis of composites to be completed at the lower sintering temperature and the shorter sintering time, resulting in a fine Ti-6Al-4V matrix and ultra-fine TiB whiskers. The fine matrix microstructure made the sliding ability of the grain boundary increase, which in turn increased the deformability of the matrix, thus reducing the possibility of damage. The ultra-fine reinforcements made it easier to release the stress and relieve the stress concentration, which could coordinate the deformation more effectively. These reasons make the alloy have good deformation ability.

**Figure 8:**
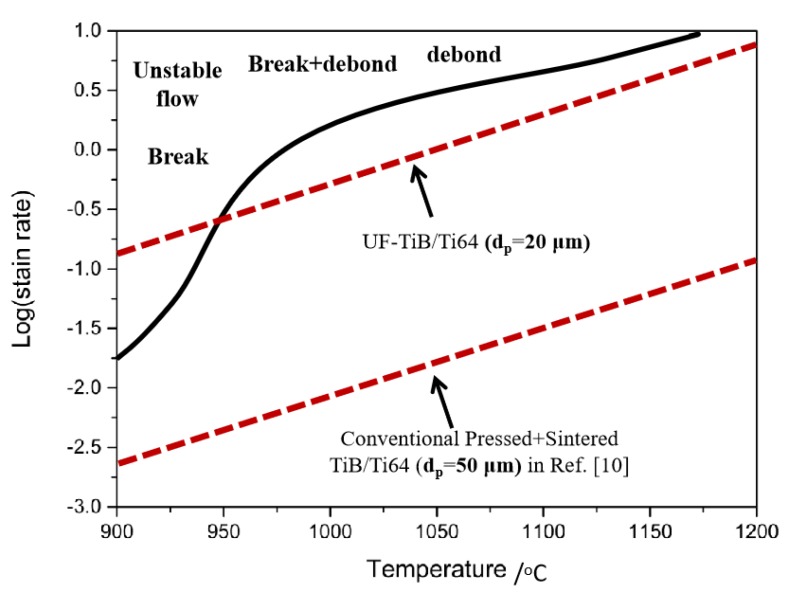
Instability map for hot working of the UF-TiB/Ti-6Al-4V composite. The critical strain rates for *p* = 16 μm of this UF-TiB/Ti-6Al-4V composite, and for *p* = 50 μm of the conventional pressed + sintered TiB/Ti-6Al-4V composite in Ref. [10].

Assuming that the whiskers were rigid and that the stress could only be released through elastic deformation and diffusion, Koeller and Raj [31] put forward a model for predicting the critical strain rate for stable deformation. The model was given as follows:

(10) $$\varepsilon_{c}=118\frac{\left(1-\nu \right)\left(1-2\nu +2/\pi \right)}{{\left(5/6-\nu \right)}^{2}}\frac{G\Omega }{k_{B}T}\frac{f_{\nu }\delta D_{B}}{p^{3}},$$

where *G*, *ν*, Ω and *δD_B_* represent the shear modulus, the Poisson rate, the atomic volume and the product of the grain boundary self-diffusion coefficient and the interface width, respectively, *p* and *f_v_* are the length and volume fraction of the TiB whiskers, and *k_B_* represents the Boltzmann constant. The parameters used to calculate the *ε_c_* were given as follows [10]: *ν* = 0.27, *f_v_* = 0.15, *k* = 1.38 × 10^−23^ J K^−1^. *p* was the largest length of the whisker (~16 μm). *G*, Ω and δ*D_c_* were 4.36 × [1 − (*T* − 300) × 6.2 × 10^−4^] × 10^10^ Pa, 1.76 × 10^−2^ nm^3^, and 8.6 × 10^−10^ exp(−150 kJ mol^−1^/*RT*) m^2^s^−1^ for the *α* titanium and 2.05 × [1 − (*T* − 300) × 2.58 × 10^−4^] × 10^10^ Pa, 1.81 × 10^−2^ nm^3^, and 1.9 × 10^−8^ exp(−153 kJ mol^−1^/RT) m^2^s^−1^ for the *β* titanium, respectively. Equation (10) relies heavily on the distribution and the size of the TiB whiskers. Considering that the distribution of the enhancement phase in the matrix was uniform, it was then applied to the calculation of the largest size of the TiB whisker. Figure 8 shows the critical strain rates at various temperatures for *p* = 16 μm of the UF-TiB/Ti-6Al-4V composite calculated using Equation (10). If the applied strain rate exceeded *ε*_c_, the composite would undergo fracture. The results of the calculation were in good agreement with the results of the processing map. The critical strain rates for *p* = 50 μm of the conventional pressed + sintered TiB/Ti-6Al-4V composite [10] were also calculated using Equation (10), as shown in Figure 8. The critical strain rates of the conventional pressed + sintered TiB/Ti-6Al-4V composite were apparently lower than that of the UF-TiB/Ti-6Al-4V composite, which further proved that the UF-TiB/Ti-6Al-4V composite had a higher deformability. This was mainly due to the fine grain size of the TiB whisker. This calculation model was performed via relaxation by diffusion only. In the high temperature deformation process, DRV and DRX also occurred, which resulted in a larger strain rate than the calculated value. Thus, the calculated value used to verify the accuracy of the processing map was reliable.

## 5. Conclusions

The hot deformation behavior of the P/M TiB/Ti-6Al-4V composite reinforced with ultra-fine TiB whiskers was studied using a high temperature compression experiment. The processing map was built to evaluate the deformability, and the microstructural evolution during deformation was studied. The main conclusions are as follows:

1. A UF-TiB/Ti-6Al-4V composite is prepared by the spark plasma sintering method. The grain size of the matrix is approximately 5–12 μm, the length of the TiB whiskers is approximately 5–20 μm, and the diameter is 0.1–2 μm.
2. The stable deformation of the UF-TiB/Ti-6Al-4V composite occurs at the conditions of 900–1200 °C/0.001–0.01 s^−1^. The optimum hot working condition is 900 °C/0.001 s^−1^, with the deformation mechanism of dynamic recrystallization (DRX).

The unstable deformation occurs when the strain rate is higher than 0.01 s^−1^, where the failure modes include adiabatic shear deformation, TiB breakage and TiB/matrix debonding.

The deformability of the UF-TiB/Ti-6Al-4V composite is much better than that of the traditional casted and pressed + sintered TiB/Ti-6Al-4V composites, which are typically reinforced with coarse-grained TiB whiskers. The high deformability is primarily attributed to the ultra-fine reinforcements, which can coordinate the deformation more effectively. The fine matrix microstructure also has a positive effect on deformability because the fine matrix microstructure can improve the grain boundary sliding.

## Figures and Tables

**Table 1:** Characteristics and content of raw powders.

Powders	Mass Percentage wt. %	Average Particle Size (μm)	Oxygen Content (wt. %)	Preparation ^a^
Ti-6Al-4V	89.7	11 ± 3.29	0.32 ± 0.025	GA
Ti	4.1	39 ± 2.16	0.25 ± 0.022	HDDH
TiB_2_	6.2	3.8 ± 0.59	0.39 ± 0.017	RO

^a^ GA, gas atomization; HDDH, hydride-dehydride; RO, reduction of oxides.
